# Patient perspectives on cancer care during COVID-19: A qualitative study

**DOI:** 10.1371/journal.pone.0306035

**Published:** 2024-07-11

**Authors:** Krista Y. Chen, Olivia Stanford, Jennifer A. Wenzel, Robert L. Joyner, Adrian S. Dobs

**Affiliations:** 1 School of Medicine, Johns Hopkins University School of Medicine, Baltimore, Maryland, United States of America; 2 Office of Community Outreach and Engagement, Sidney Kimmel Comprehensive Cancer Center, Johns Hopkins University School of Medicine, Baltimore, Maryland, United States of America; 3 School of Nursing, Johns Hopkins University School of Medicine, Baltimore, Maryland, United States of America; 4 Sidney Kimmel Comprehensive Cancer Center, Johns Hopkins University School of Medicine, Baltimore, Maryland, United States of America; 5 Richard A. Henson Research Institute, TidalHealth Peninsula Regional, Salisbury, Maryland, United States of America; 6 Department of Medicine, Johns Hopkins University School of Medicine, Baltimore, Maryland, United States of America; Public Library of Science, UNITED KINGDOM OF GREAT BRITAIN AND NORTHERN IRELAND

## Abstract

**Purpose:**

The COVID-19 pandemic posed unique challenges to cancer-related care as health systems balanced competing risks of timely delivery of care and minimizing exposure to infection in a high-risk, immunocompromised patient population. This study aimed to better understand how pandemic-related factors affected the patient experience of cancer care during this time.

**Methods:**

We conducted fifteen semi-structured interviews with adults from rural counties in Maryland who were diagnosed with and/or actively treated for cancer at the TidalHealth healthcare network between January 2020 and October 2022.

**Results:**

Interviews from fifteen participants were analyzed. Two major themes emerged including COVID Impact on Care, and COVID Impact on Mental Health. Subthemes under COVID Impact on Care include Staffing Shortages, Hospital Regulations, Visitation, Importance of Advocacy, and Telehealth Utilization, and subthemes under COVID Impact on Mental Health include Loneliness, Support Networks, and Perceptions of COVID and Personal Protection. Overall, participants described positive care experiences despite notable delays, disruptions to continuity of care, difficult transitions to telemedicine, visitation policies that limited patient support, increased mental health struggles related to social distancing measures, and greater desire for patient advocacy.

**Conclusion:**

Our findings reveal significant impacts of the COVID-19 pandemic on experiences of cancer treatment and survivorship in a more vulnerable, rural patient population with lower healthcare access and income level. Our findings suggest areas for targeted interventions to limit disruptions to quality care in future public health emergencies.

## Introduction

Cancer has been one of the top two leading causes of death in the last 75 years [1]. In 2023, the United States had an estimated 1.9 million new cases and over 600,000 cancer-related deaths [2]. However, the burden has not fallen equally on all individuals as major disparities along racial, ethnic, socioeconomic, and geographic lines can greatly impact cancer survival rates and overall health outcomes [3–5]. Maryland is one such state with great variation in healthcare access as it contains a wide spectrum of highly urbanized and rural settings. Between 2016–2021, Maryland had similar age-adjusted incidence rates compared to the national average, but the rural counties along the Eastern Shore of Maryland had significantly higher age-adjusted cancer incidence rates with a nearly 20% increase in cancer incidence rates compared to that of the rest of the state [6].

While disparities in cancer care have always existed, the COVID-19 pandemic magnified and exacerbated many longstanding inequities as the unprecedented burden on healthcare systems during the COVID-19 pandemic led to significant disruptions across the cancer care continuum [7–12]. Cancer is a complex and multifaceted disease that requires effective coordination and integration of multiple healthcare services including oncology, surgery, radiology, pathology, and palliative care into highly personalized treatment plans [13, 14]. Small delays or fragmented care may have detrimental consequences [15–17], as much literature documents the associations between increased time to initial diagnosis/treatment with decreased overall survival, decreased local control, and increased morbidity due to the physical (fatigue, anemia, cachexia), mental (fear, anxiety, social isolation) and socioeconomic (financial toxicity, lost wages, decreased employment opportunities) effects of greater disease burden [18–20].

As individuals with cancer are at greater risk of contracting COVID-19 and developing severe illness or complications due to their immunocompromised state [21–23], healthcare facilities providing care for oncologic and hematologic malignancies faced additional challenges during the pandemic in balancing the need for comprehensive, timely care while minimizing exposure to infection. Many facilities, both inpatient and outpatient, thus found adaptations to continue operations while implementing considerable changes in workflow to increase hygiene and social distancing measures [11].

Given the major disruptions to healthcare systems and the complexity of care associated with oncologic/hematologic encounters, the objective of this study was to better understand how the COVID-19 pandemic and resultant safety measures may have impacted the patient’s experience of cancer care. While a large body of epidemiological and quantitative studies have been produced in response to the pandemic, there has been a much smaller emphasis on understanding the patient perspective. Moreover, to the best of our knowledge, no existing qualitative studies focus on the rural, medically underserved communities of Maryland, a patient population that perhaps provides further insight into barriers to care, continuity, and access in more vulnerable regions. The findings from this study thus not only inform the course of current pandemic response, but also inform strategies for future public health emergencies that may limit disparities and disruptions to care.

## The current study

### Background

For this study, we used a qualitative descriptive approach to capture the experiences of patients with cancer and describe how their lives were impacted by the COVID-19 pandemic. This approach adheres closely to the participants’ own language, using little interpretation when capturing the perceptions, sensitivities, and sensibilities of the describer. It offers a flexibility that allows for an initial research question to be reformed and refined as more data are collected [24]. This approach is commonly applied towards problem identification and concept development, in this case, making it an ideal method for understanding major sentiments and challenges during a never-before-seen public health emergency and for gaining insights for future crisis planning.

All patients were recruited from the Richard A. Henson Research Institute at TidalHealth Peninsula Regional (from here on referred to as TidalHealth), a healthcare network providing primary and specialty care through clinics across Delaware and Maryland and primarily serving low income, rural populations with high concentrations of migrant workers. Eligibility included adults diagnosed with and/or treated for cancer at TidalHealth on or after January 1, 2020, and who reside in three rural counties on Maryland’s Eastern Shore: Wicomico, Worcester, and Somerset Counties. All recruitment and interviews were conducted by staff from TidalHealth and Johns Hopkins University who acted as study team members. Recruitment began in November 2021 and ended in October 2022. Interviews occurred between November 2021 and December 2022.

We invited potential participants via geographically targeted advertisements on social media platforms (i.e. Facebook) and community websites (i.e. Craigslist); by sending a secure, private message to eligible patients through the internal electronic health record system (EHR), MyChart; and by verbal mention to eligible patients by providers at TidalHealth.

Ethical approval for the study was granted by the Johns Hopkins School of Medicine Institutional Review Board (IRB00231545). All participants were provided written informed consent prior to the interview. Verbal consent was also attained at the time of interview using an IRB-approved verbal consent script, and this process was documented in an additional form with patient identifiers removed prior to recording. No relationships between the research team and the participants were established prior to the start of the study. Funding for this study was given by Climb for Hope, Inc.

### Sampling

Due to our use of public forums for recruitment, we are not able to quantify the total size of the population of interest. However, the study team contacted 299 eligible participants identified through the EPIC EHR. These eligible participants ranged from 28–97 years old and identified as 59.9% female, 40.1% male, 76.3% White, 20.1% Black or African American, and 1.7% Hispanic. This population also included patients of more than 20 different primary cancer sites; most common sites included prostate (38.3%), breast (21.7%), and lung (9.8%). The final sample size for our study was 15 people, representing 5.0% of the total population of interest.

### Data collection and analysis

Prior to the start of the study, a semi-structured interview guide [Tables A and B in S1 Table] was developed and externally reviewed by a research colleague and our methods expert. The guide was also tested within the study to ensure qualitative validity and rigor by two study team members as they conducted interviews. The guide was further reviewed several times by another study team member who acted in an advisory capacity throughout the duration of the study. All interviews were completed over Zoom or by telephone using the guide. Interviews were audio-recorded and transcribed verbatim. Security precautions were taken throughout the entire recruitment and interview process to safeguard participant anonymity and confidentiality.

Fifteen interviews were transcribed and analyzed using NVivo 12 (QSR International, 2023. Two independent team members separately coded transcripts. A consensus coding approach was used to establish intercoder agreement for all transcripts until consensus was achieved [25]. Iterative changes were made as study team members met regularly to discuss emerging themes, ensure coder consistency, and avoid coder drift. After that, two additional team members reviewed interviews and codes to further validate the coding process and ensure representative quotes, subthemes, and themes were adequately portrayed in the final interpretation of the data. When no new insights were identified from thematic analysis of participant interviews, three study team members determined sampling saturation was achieved with 17 semi-structured interviews. Two interviews were not included in the analysis due to technical difficulties but were found by the interviewers to be consistent in theme with the others.

## Results

The study cohort consisted of 15 participants, representing survivors of breast, colon, lung, and prostate cancer, the four most common cancers in the US. All patients reside on the Eastern shore of Maryland and were between 34 and 76 years at the time of interview. A majority of patients identified as White (93.3%) and female (80.0%), and most (86.7%) were in active treatment during the peak of the pandemic, between 2020–2021 (Table 1).

**Table 1:** Demographics and disease characteristics.

Variable	Classification	N (%)
Number of Patients		15
Age at Interview (years)		Mean 61.3 (Range 34–76)
Gender	Male	3 (20.0%)
	Female	12 (80.0%)
Race^a^	White	14 (93.3%)
	Black or African American	1 (6.7%)
Cancer Type	Breast	9 (60.0%)
	Colon	1 (6.7%)
	Lung	4 (26.7%)
	Prostate	1 (6.7%)
Treatment Status	Active Treatment^b^	13 (86.7%)
	Remission	2 (13.3%)

^a^Race categorizations identified by patient

^b^This refers to active treatment during 2020–2021, the peak of the pandemic

Two main themes and eight subthemes were identified through analysis of participants’ accounts of cancer care and survivorship during the COVID pandemic (Table 2).

**Table 2:** Themes and subthemes.

Themes	Subthemes
COVID Impact on Care	Staffing Shortages
	Hospital Regulations
	Visitation
	Importance of Advocacy
	Telehealth Utilization
COVID Impact on Mental Health	Perceptions of COVID and Personal Protection
	Loneliness
	Support Networks

### Theme 1: COVID impact on care

Overall, participants felt positively about the care they received during the pandemic despite variations in wait times and regulations. For five participants (33.3%), the pandemic seemed to have little to no effect on care, as all appointments and treatments were scheduled in a timely manner.

“I could say that actually COVID-19 hasn’t had any impact on my cancer care. I’ve been able to maintain all my appointments and basically all my treatments.”–LPTH-16

“I don’t think I ever had to wait more than once or twice in the entire 2–3 months of my treatment. More than 5 or 10 minutes because they were slightly backed up, but there was nobody else in the waiting room.”–LPTH-5

Positive sentiments were also expressed by the other ten participants (66.7%) who did experience delays, as they understood the prioritization of patient safety in challenging, new circumstances.

“I don’t think they could have done anything differently. I think they erred on the side of caution, which is exactly what you need to do in the pandemic.”–LPTH-1

Minor reasons for delay included waiting as providers were occupied with other patients, withholding COVID vaccination due to unknown interactions with current oncology treatment, or holding off on procedures due to COVID positive status. Notably, one participant waited six weeks for a diagnostic biopsy because she continued to test positive for COVID following infection.

“[COVID] had a big impact. I was not able to get prompt treatment because they wanted to do a right upper lobectomy, but they were afraid to do it while I had COVID understandably. So, finally, they decided, ‘the heck with it. We’re going to do the surgery because we can’t wait. It’s been six weeks’… All this time I had never seen a doctor because they weren’t seeing patients, so it was all done over the phone.”–LPTH-1

#### Staffing shortages

The most notable delays were often related to individuals transferring care. Five participants (33.3%) noted changes in staffing and/or difficulty ascertaining consistent care during the pandemic. One participant reported not being able to find a primary care physician for a year and a half after moving. Two others commented on difficulty maintaining continuity of care, with one participant transitioning through four oncologists due to physician relocation, quitting, or maternity leave–including one month where they were without a provider completely.

“My doctors kept quitting, so I kept changing doctors… then I got a PA… now I’ve got a doctor who was previously from [healthcare institution] treating me because my doctor is on maternity leave… the doctors are leaving like crazy, you know, you get one and then next thing you know, you got a different one.”–LPTH-2

“I had to stick with my primary care provider [from before the move] because I couldn’t find anybody here. Nobody was taking on new clients, because they barely had enough resources to take care of their existing patients.”–LPTH-6

Two participants (13.3%) observed workplace shortages that seemed to increase strain and patient load on their providers.

“One day, [my oncologist] said he needs roller blades. They’re understaffed, I’ll put it that way. In the oncology department, I could tell they’re understaffed as far as doctors, and I even heard that mentioned by some of the workers. But I felt like we all got the care that [we needed]… Unfortunately, [the oncologist] left the practice.”–LPTH-14

“That place is not running well. Well, I think they’ve really got short staff. Getting messages through and that kind of stuff… I haven’t been happy. I think they are just overloaded because so many people have left.”–LPTH-12

#### Hospital regulations

All participants (100%) noted an increase in regulations, some of which led to increased wait times, at their respective healthcare facilities. These included COVID screening and questionnaires prior to encounters, mandatory masking, increased cleaning measures, social distancing in waiting rooms, and increased stations for hand sanitizing. Participants generally expressed appreciation for these precautions, observing that procedures were beneficial for patient safety. Conversely, other changes were received poorly. Notably, some ancillary staff and services were also removed, with one participant noting an increase in stress due to removal of valet parking.

“They could park for me because I’m really fatigued, very, very fatigued, and so for me to park out in the parking lot and walk up to the door and all that kind of stuff, I just really enjoyed the valet service. Now my husband drives me to the appointments… Without the valet service, that turned into a more stressful time for me because I have to rely on him.”–LPTH-2

#### Visitation

Visitation was uniquely identified as a regulation that impacted participants’ experiences with healthcare. Visitation policies changed sharply regardless of appointment type, with either no visitors or at most one visitor allowed during chemotherapy sessions, oncologic or primary care encounters, outpatient procedures, or before and after major surgeries. For encounters with no visitors, participants commented on the inability of designated caregivers to communicate or coordinate with them from outside of the hospital during procedures and extended stays. For encounters with one visitor, participants noted difficulty in having to choose one family member who perhaps had greatest health literacy to accompany them, while others could not participate in their support.

“After my surgery, you weren’t allowed to have people in there. That was really tough… During the chemo treatment, you know, nobody was allowed back there.”–LPTH-8

“It was more from the external of my sister as the ride, the caregiver, to be able to get what she needed to be able to assist me.”–LPTH-6

“There could only be one designated visitor for my entire stay. [My four children] wanted to take turns, and they couldn’t.”–LPTH-12

#### Importance of advocacy

The reduced visitation and support staff in the hospital impelled participants or their caregivers to be better advocates to ensure receipt of the best care possible and ensure the most apt treatment decision-making during this stressful period.

“[My daughter] had to stay in the car, and you know, you go into the room by yourself, and I have a tendency not to go into detail on things whereas [my daughter] wants to make sure she understands something extremely clear… It’s better to have someone in the room with you as you hear different things, and she was always good about pressing for answers.”–LPTH-11

"Well, you had to be your own advocate, you know, it’s people my mother’s age who don’t think to look, or they don’t know what they want to look for. I’m not necessarily saying I’m looking for a specific answer. I’m just looking for enough information to help make a decision.”–LPTH-6

Three participants (20.0%) noted that they wanted to rely more heavily on nursing staff for support and advocacy.

“I wish I had [more attentive] nurses when I was recovering from the double mastectomy because I just felt like I really wasn’t checked on or heard, and like I said, there wasn’t anybody. You know, she wasn’t allowed to be with me, my sister… I was in the surgery for like 6 hours. I had the anesthesia. I just can’t self-advocate for myself, to, you know, make it clear what I actually need or want.”–LPTH-8

#### Telehealth utilization

When engaging in cancer care, less than half (6/15, 40.0%) of participants utilized telehealth. Of these, two had positive experiences, while four had neutral or negative experiences. A few participants expressed confusion regarding the rapid transition to telemedicine without explanation that it would supplement continued in-person care.

“I think that what is confusing about the telehealth is that there’s not really an explanation of like what this is or how it’s supposed to look, so like they just say, ‘we’re doing this telehealth’… There was no ‘here’s what we do during telehealth appointments,’ or ‘here’s things that you can expect.’”–LPTH-1

Positive experiences were often attributed to speed and convenience, especially for follow-up and check-in encounters with decreased urgency.

“The best part of it was the saving of the time and the driving because that’s, that’s really exhausting. And so early in my recovery, I didn’t have a lot of energy. So, you know, I, I just don’t know how I would have done five hours of driving.”–LPTH-1

A majority of participants who experienced telehealth felt that it was more difficult to connect with providers, requiring greater intentionality on the provider’s behalf or greater advocacy on the patient’s behalf.

“I just feel that they’re really impersonal because they’re only seeing your face and you’re only seeing their face. For a whole picture of how somebody’s health is doing, you have to be there in person, you know. Do they twitch, you know? Are they shaking their leg? Are they doing this, and are they doing that, you know?”–LPTH-13

“You get more of a sense of sympathy. Because when you’re with somebody, you can see the body language and the facial expressions better than, you know, over Zoom.”–LPTH-1

Importantly, participants also noted practical limitations stemming from limited access to technology in terms of equipment and user knowledge.

“Every morning, I will go get my grandson… I have an old computer so I couldn’t get Zoom on that… It’s probably better for younger people who are less technologically challenged than I am.”–LPTH-2

“My husband is maybe about 60–65% deaf, so he can’t do telehealth… He needs me to be with him and walk through things with him. So that’s another stress factor… It would’ve been very hard for him to communicate.”–LPTH-6

### Theme 2: COVID impact on mental health

Many participants (9/15, 60.0%) expressed times of acute or chronic distress affecting their mental health, particularly during the early phases of diagnosis, immediately after treatment and procedures, or after complications. Of these, one had previously established longitudinal mental healthcare. Three participants initiated what turned into long-term mental healthcare that continued through the time of interview. For many, these feelings were reported in reference to their cancer course, and it is unknown the extent to which they may have been exacerbated by the pandemic. Overall, there were reports of depressive symptoms, suicidal ideation, and anxiety stemming from a combination of uncertainty about the situation and disease; change in self-concept, ability, or role; fear; and loneliness.

“I was very, very, very upset. And I just didn’t feel like I could go on any farther… When I lay down at night, I wished I didn’t wake up in the morning. I just couldn’t handle it anymore.–LPTH-2

“It’s just frustrating… It affects your muscles and your body and you know, I can’t do things I used to be able to do.”–LPTH-11

#### Perceptions of COVID and personal protection

Ten participants (66.7%) expressed greater sense of urgency towards personal hygiene measures and greater fear of contracting COVID due to their immunocompromised states. Many reported isolating at home for long periods than the general population, past the point when most other individuals began returning to pre-COVID life.

“Even the common cold could give me harm. The flu could give me real harm. Pneumonia could give me even more harm, and then COVID could do me even more harm.”–LPTH-15

“I’m afraid to go out with COVID. And now with every variation of it, omicron or whatever the heck it’s called. You know, I wear a mask every time I go out because I don’t think my lungs are able to handle it if I caught COVID.”–LPTH-13

“So I felt pretty safe here at home because I never went anywhere. You know my husband and I were careful more, we went out and we did wear our masks and we were real careful with who we were around.”–LPTH-3

For many, maintaining isolation required working from home. However, at least one patient noted an inability to work for 19 months as he could only work in person.

“Once I wanted to go back to work, both [my physicians] were like you cannot be around humans. I live alone, I’m single, and I went from 2019 up until I think 2021 before I was allowed to go back to work.”–LPTH-11

These measures also forced participants to do a cost-benefit analysis of various social luxuries. For example, some stopped participating in hobbies, going to the gym, or attending community groups to avoid any risk of exposure to COVID.

“I love going to live heavy metal rock concerts, but I haven’t been to one. I stay away from crowds. I stay away from them. But I was like, paranoid to go out and do anything for a while.”–LPTH-12

In more extreme cases, these measures led to antagonism of individuals who may have put them at greater risk, including family and community members who were not vaccinated.

“I get angry at the people that don’t get vaccines, don’t understand how they can be so stupid.”–LPTH-4

“It’s been pretty rough. I lost [two sisters and a brother] within one year. I missed seeing my sister before she passed. And I couldn’t go see [my brother] because he refused to have the COVID shot… You know other than that, that’s basically the worst impact we’ve had–not being able to be around family, you know, like we should.”–LPTH-3

#### Loneliness

Six participants (40.0%) reported that these social isolation measures directly amplified feelings of loneliness, severely impacting mental health.

“I have to be very, very, very careful. You know, I just can’t be around crowds, can’t be around sick people, so on and so forth. And it was exponentially more important during that time… It started to become a problem. Just living by yourself. You’re not allowed to be around humans for about 19 months.”–LPTH-11

“I haven’t been working, and I don’t have any kids in the house, and I’m not married or anything else… Before that I worked in [my job]. So all of a sudden, now I’m here by my lonesome. It’s secondary to the cancer because… I had to kind of stop running around like a frantic person, and all of a sudden, you know, I started working on myself.”–LPTH-12

#### Support networks

Across all participants with both acute and long-term impacts on mental health, participants reported a desire for supportive services from healthcare staff to alleviate distress.

“Honestly, I will have to say those nurses are God sent. They are. They’re really amazing… I really think it helped out mentally for me a lot, especially because you can’t have anybody with you.”–LPTH-8

“[The social worker] was only supposed to see me three times, but she saw me way more than that. Anyways, that was really helpful.”–LPTH-12

Many participants also expressed the importance of outside networks in maintaining their mental health and supporting their treatment course and symptom management. These included family, neighborhood groups, faith-based communities, and cancer survivorship groups.

“Without [my family], I don’t think I’d have held it together. I’ve always been one to not want to ask for help… [Now], I would definitely ask for support. More support sooner than I did. I’m used to being on my own for this many years, and it’s like you don’t want to ask anybody because you don’t want to put anybody out. But a support system, you can’t go without.”–LPTH-13

In reference to a cancer survivorship group: “…just knowing that they were there if I had questions… one of the counselors there on the phone, she’s called and checked in on me, and then she leaves me messages. I call her back and let her know where I am and that I’m doing okay.”–LPTH-17

“I’ve met a group of people in the neighborhood who would kind of have a social walking group every day at 4 o’clock. I tried it. That was good therapy.”–LPTH-5

“I go between [my] kids and a great community of friends… they were bringing me stuff, and so I really didn’t have to go out… I have never felt more love then when they were here with me.”–LPTH-12

Overall, participants described major impacts to cancer care and major effects on mental health during COVID-19. Although all recounted generally positive sentiments, there were some noted delays to care, frequent changes to staff, difficulty establishing continuity of care, hospital regulations that increased procedure times and decreased access to useful services, and visitation policies that limited patient support during encounters. Implementation of telehealth was found to be both a positive and negative force with increased convenience but challenges to accessibility and establishment of therapeutic relationships. Moreover, participants described depression, loneliness, and anxiety related to social isolation and personal protection measures, emphasizing a greater need for advocacy and support systems during this time.

## Discussion

The goal of this study was to explore the lived experience of cancer treatment and survivorship during the COVID-19 pandemic. Qualitative analysis of semi-structured interviews was conducted with 15 adults from the Eastern shore of Maryland, a primarily rural and low-income region with low healthcare density and a population often disenfranchised from research being done in academic medical centers [26, 27]. This analysis highlighted major changes to cancer care models that still allowed for positive care experiences despite staffing changes, loss of care continuity, distressing hospital regulations, and difficult transitions to telemedicine, as well as major changes to patients’ lives that led to increased concern for personal safety and increased demand for social support, patient advocacy, and mental health services.

The COVID-19 pandemic led to dramatic shifts in healthcare as new regulations were imposed to ensure safety of all patients and workers and great levels of burnout led to a significant number of individuals leaving the workforce. Much of the literature from this period documents staffing shortages [28–30] and reduced healthcare utilization [31, 32], leading to decreased delivery in areas across the cancer continuum including screening, imaging, pathologic reporting, surgical treatment, radiation treatment, psychosocial care. Accordingly, participants from our cohort noted delays to procedures and treatments and having to rotate through multiple oncologists as well as difficulty finding new oncologists and primary care physicians. Although this study was not designed to establish direct impacts of the pandemic on health outcomes, prior research suggests that significant delays in cancer care may increase morbidity and mortality [33, 34].

In understanding the context of these shortages, it is important to note that these participants may have been biased by integration into the healthcare system prior to the pandemic. Some participants noted a lack of other patients in clinics. While this observation may have been due to social distancing measures, this pattern is also reflected in previous literature as screening and diagnosis were amongst services most greatly impacted during COVID-19 [12, 35, 36]. One study estimates that 40,000 fewer individuals started cancer treatment in the UK alone during the pandemic [37, 38]. As we revert to prior levels of screening and diagnosis, health systems may expect to see increased burden and complexity due to increased late-stage disease, thus underlying the importance of future mitigation strategies to minimize interruptions and delays.

A major element of the COVID-19 pandemic is the rise of telemedicine. Although telehealth has been shown to be especially beneficial for rural residents, such as the participants in this study, only 6/15 participants utilized the modality throughout their care. This may be due to the complexity of care associated with oncological encounters, requiring complex decision-making and more frequent testing and making it more suitable for in-person care [39]. Among those who used telehealth, experiences aligned with existing literature [40–43]. Advantages described include time saved, lower cost, and conserved energy for sicker patients. Disadvantages primarily stemmed from greater difficulty connecting with providers and decreased accessibility due to disability or technological challenge. Technological challenges may be over-represented in our cohort as it is mainly comprised of older patients in counties that rank among the lowest in educational attainment in Maryland. Furthermore, large areas of the Eastern Shore have decreased telecommunication infrastructure, potentially limiting access for those with technical proficiency. Overall, as telehealth appears to be a mainstay of the post-pandemic world, our findings necessitate further studies to optimize its utility and ensure reliable and equitable integration into standard healthcare practice, targeted especially in areas of lower digital access. Interventions may include more detailed guidelines for providers and patients alike on effective use of telehealth or expanded insurance coverage for items necessary for telehealth services including video cameras and microphones.

Another prominent theme highlighted by this study was the experience of loneliness. Risk for loneliness is higher at baseline for cancer patients and survivors, even after controlling for prior depression, anxiety and other mental health factors, and it is associated with health risks including poorer cognitive function, decreased immunity, and increased risk for recurrence and mortality [44–47]. During the COVID-19 pandemic, patients noted how various factors aimed at increasing their safety ultimately led to increased morbidity due to serious mental health effects. Participants expressed marked levels of anxiety and uncertainty while going through emotionally difficult encounters or procedures without caregivers or family members due to limited visitation. These increased levels of anxiety extended outside the hospital setting as concern for personal safety contributed to further isolation measures in the personal sphere–ranging from giving up community groups and hobbies to refraining from employment or attending funerals of loved ones. Overall, although some level of safety precaution will always be necessary, healthcare systems should make it a priority to mitigate the detrimental effects of isolation and identify strategies to allow for social and emotional support during encounters. Healthcare systems should also look to improve early assessment or screening for all individuals undergoing cancer treatment and proactive referral to mental and behavioral health services for vulnerable patient populations.

Lastly, a major factor associated with all of these findings, including reduced telehealth experiences, loss of continuity due to staffing changes, visitation policies, and lack of social support networks, was that patients observed a greater need for self-advocacy. This emphasizes the need for providers to be more attuned to the communication styles of patients and for systems such as health navigation programs and patient advocates to be available and accessible during future emergencies.

### Limitations

This qualitative assessment was subject to a number of limitations. First, the sociodemographic makeup of our cohort as well as the smaller sample size may limit the generalizability of our findings as they may not be representative of all cancer survivors in the United States. Indeed, the breakdown of our patient population was comprised primarily of White (93.3%) and female (80.0%) individuals diagnosed with breast cancer (60.0%) from a more rural, under-resourced community with low educational attainment and income level lower than the national average, and our data was not able to support stratification of results by cancer type or any other demographic variable. The study cohort may not have been completely representative of the total, eligible population recruited at TidalHealth, with higher proportions of White, female individuals suggesting some level of selection bias. All interviews were conducted over Zoom, excluding individuals without access to or proficiency with internet. As interviews were conducted between 2021 and 2022, a year after the start of the pandemic, this may affect accuracy of participant recollection. All results were self-reported and subject to social desirability and recall bias.

## Conclusions

Our study sheds light on positive and negative experiences of cancer treatment and survivorship during the COVID-19 pandemic. While the focus of this study was on experiences during COVID-19 specifically, this enhanced understanding of oncologic patient perspectives could potentially apply to other similar and unpredictable disruptions to healthcare utilization, workflow, and access as well as disruptions to personal life that may occur during other public health emergencies. We highlight multiple areas that hospital systems may consider in future crisis planning to aid the transition from active outpatient models to hybrid models of care and support networks to recapitulate the wide range of social and medical services required by patients.

## Supporting information

S1 Table Interview guide. (DOCX)
