# Supplementary material for: Patient perspectives on cancer care during COVID-19: A qualitative study
Source: PLoS One. 2024 Jul 11;19(7):e0306035. doi: 10.1371/journal.pone.0306035 (PMC11238955; doi:10.1371/journal.pone.0306035)
Supplement: S1 Table — (DOCX) [file pone.0306035.s001.docx]

**Table A. Interview Guide Part 1.**

| Domain | Example questions |
| --- | --- |
| Opening question/  explore respondent  characteristics | 1. Tell us about your cancer journey.    1. When were you diagnosed?    2. When did you start and/or end treatment?    3. What was your treatment like?    4. What sort of do’s and don’ts, or precautions did your providers talk to you about when you were diagnosed/starting treatment? |
| COVID-19 effects on health and life | 1. How have you been doing during the pandemic? [Tell us about your health throughout the pandemic?]    1. (They can talk about pre-existing conditions, chronic diseases, etc.) 2. Have you ever had COVID-19?    1. If so, what was it like for you?    2. What experiences do you have with long-term COVID symptoms (also known as long COVID)? 3. What health concerns did you have due to COVID-19? 4. How did you protect yourself in the pandemic? 5. What did your provider tell you specifically about COVID? |
| COVID-19-related  Issues on cancer care | 1. What impact has COVID-19 had on your cancer care? (For example, delays in treatment, finding a provider, visitation or support, change in hospital services, etc.)?    1. How are those impacts affecting your cancer treatment?    2. **If no impact, refer to Table B of interview guide.** |
| Mental Health | 1. How was your mental health during the pandemic? [How have you been feeling?] 2. How did your care team address your mental health concerns? 3. How did your cancer diagnosis/treatment impact your mental health? |
| Care coordination | 1. What are your expectations for cancer care vs other types of care?    1. What do you expect from your providers? (For example, time, communication, support, knowledge of resources, etc.)    2. What previous care encounters have you had (in general)? 2. How has your cancer team addressed COVID-19?    1. What is your team doing well?    2. How could your care during COVID-19 be better?    3. How has your care changed due to the pandemic? 3. What has stayed the same?    1. Tell me about your experiences with telehealth during the pandemic.    2. How have telehealth visits impacted your cancer care? What is going well with telehealth visits?    3. What could be better about telehealth visits?    4. **If they say no experience w/ telehealth: What would make telehealth better? What would you like from a telehealth visit?** |
| Closing Question (Ask one/combination) | 1. Looking back, what is your biggest takeaway from the pandemic? 2. What is ONE thing you would tell your past self, regarding COVID and your cancer? |

**Table B. Interview Guide Part 2.**

| Domain | Example questions |
| --- | --- |
| Mental Health | 1. How was your mental health during the pandemic? [How have you been feeling?] 2. How did your care team address your mental health concerns? 3. How did your cancer diagnosis/treatment impact your mental health? |
| Care coordination | 1. What are your expectations for cancer care vs other types of care?    1. What do you expect from your providers? (For example, time, communication, support, knowledge of resources, etc.)    2. What previous care encounters have you had (in general)? 2. Let’s talk about your cancer care team.    1. What did your team do well?    2. What could they have done better? 3. Tell me about your experiences with telehealth/phone visits during the pandemic.    1. How have telehealth visits impacted your cancer care?    2. What is going well with telehealth visits?    3. What could be better about telehealth visits?    4. **If they say no experience w/ telehealth: What would make telehealth better? What would you like from a telehealth visit?** |
| Closing Question (Ask one/combination) | 1. Looking back, what is your biggest takeaway from the pandemic? 2. What is ONE thing you would tell your past self, regarding COVID and your cancer? |
